# Minimal intervention dentistry for treating primary teeth: a survey study among members of the Israeli Society of Paediatric Dentistry

**DOI:** 10.1007/s40368-024-00977-5

**Published:** 2024-12-09

**Authors:** M. Moskovitz, T. Elkesslasy, A. Shmueli, E. Halperson, D. Ram, A. Fux-Noy

**Affiliations:** 1 Department of Pediatric Dentistry, Hadassah Medical Center, Faculty of Dental Medicine, Hebrew University of Jerusalem, P.O.B. 12272, 9112102 Jerusalem, Israel; 2 Faculty of Dental Medicine, Hebrew University of Jerusalem, Jerusalem, Israel

**Keywords:** Minimal intervention, Primary teeth, Dental treatment, Children

## Abstract

**Purpose:**

To assess the attitudes of dentists in Israel to minimal intervention on primary teeth.

**Methods:**

For this cross-sectional study, data were accessed from questionnaires that were completed anonymously by members of the Israeli Society of Paediatric Dentistry. For four clinical scenarios, the respondents were asked to describe the stage at which they would intervene, the type of preparation they would utilize, and the restorative materials they would use. The scenarios included proximal and occlusal caries in the primary molar and buccal and proximal caries in the primary maxillary incisor.

**Results:**

Forty-six dentists completed the questionnaire. Forty-one (89%) cited that they would intervene in stages 3 and 4 (of 6) of proximal caries in the primary molar; 34 of these stated that the cavity preparation would be of the proximal box type. For occlusal caries, 37 (80%) stated they would intervene in stages 2 and 3 (of 5); 31 of these would only remove the carious lesion. Thirty-three (72%) of the respondents stated they would intervene in stage 3 (of 4) of buccal caries; 31 (67%) stated they would intervene in stage 2 (of 4) of proximal caries in the primary maxillary incisor.

**Conclusion:**

The study reveals that specialists and non-specialists dentists in Israel use minimally invasive dentistry to treat children, either by intervening at a later stage of tooth decay or by employing conservative techniques to restore teeth. This aligns with the philosophy of minimal intervention.

## Background

Early childhood caries (ECC) is still a prevalent disease among preschool-aged children, regardless of their country's level of development. However, worsening can be prevented by the provision of diet advice, the promotion of good oral hygiene practices, and the utilization of preventive measures such as topical fluoride. Management of ECC often requires restorative treatment at an early age (Berkowitz 2003; Anil and Anand 2017). Those treatments require local anesthesia and the use of a rotatory handpiece, which can initiate negative behavior in children (Santamaria et al. 2015). General anesthesia, deep sedation, or moderate sedation is sometimes required, due to the difficulty of young children to cope with the extensive treatment procedures (Berkowitz 2003; Anil and Anand 2017). Restorative dentistry has little to no long-term impact on oral bacteria like *S*. *mutans*. Hence, the clinical outcomes for treating ECC are poor. Many treated children experience new caries lesions within 6–24 months after initial dental treatment (Berkowitz 2003). The approach of restoring primary teeth has been questioned (Innes et al. 2013; Hansen and Nyvad 2017), as a large proportion of primary teeth with untreated carious lesions exfoliate without showing any symptoms (Hu et al. 2013). Minimal intervention dentistry is an alternative approach for managing dental caries, which focuses on prevention and early detection rather than surgical intervention. The core principles of minimal intervention dentistry can be summarized as Recognition of disease contributory factors; Re-orientation of contributory lifestyle factors; Remineralization of all lesions, visible and not visible, cavitated and non-cavitated; Repair, where no other solution is possible; and Review of the child, their oral health, and their life environment. These core principles of minimal intervention dentistry enable comprehensive patient/family assessment, with early diagnosis of carious lesions, reliable caries risk assessment, implementation of effective preventive measures, and minimally harmful restorative care (Innes and Manton 2017).

Carious lesions should be repaired when cavitation results in a non-cleanable lesion, weakens the tooth structure or compromises esthetics. In the sealing-in technique, the cariogenic biofilm is managed by its being sealed from the oral environment. Carious lesions isolated from the oral environment do not progress if the seal is effective and durable over time. Sealing-in strategies include the use of fissure sealants to arrest carious lesions in pits and fissures that did not progress past micro-cavitation, sealing with a preformed metal crown (Hall Technique) with no carious tissue removal, and sealing with an adhesive restorative material after selective (partial) carious tissue removal (Innes and Manton 2017). Based on a survey of dentists, Laske et al. (2019) concluded that a global shift toward less-invasive methods for treating caries lesions had not occurred, neither in the initiation of treatment nor in the preparation techniques used. However, in some countries, changes over time could be assessed, indicating that more dentists postpone interventions until lesions progress to a deeper level. The researchers found that dentists worldwide tend to intervene operatively too early for caries though this differs across countries. A worldwide shift was observed in the restorative material applied as the composite resin has almost completely replaced amalgam for restoring primary caries lesions.

Several studies (Espelid et al. 1985, 2001; Laske et al. 2019) described the conservative choices made by dentists regarding treatment thresholds and restorative techniques in permanent teeth. The current study aimed to investigate dentists' choices in treating primary teeth. The main objective was to assess the attitudes of dentists in Israel to minimal intervention on primary teeth. To this end, we inquired regarding the stage at which dentists would choose to intervene invasively, and the type of intervention and the material they would use. The secondary objective was to explore associations between the characteristics of dentists and their choices.

## Materials and methods

### Study population and design

This cross-sectional study was based on the analysis of data accessed from questionnaires that were distributed online to members of the Israeli Society of Dentistry for Children (ISDC). The members of ISDC are specialists and residents in pediatric dentistry and general practitioners who treat children. The study inclusion criteria were dentists who were members of ISDC in 2022, and who filled and submitted questionnaires.

### Study tool

The research tool used was an anonymous pre-coded questionnaire that was developed by Laske et al. (2019) and modified by our research team for use in pediatric dentistry. The modifications included transforming presentations on permanent molars to primary molars and adding two presentations of caries lesions in primary maxillary incisors. The questionnaire consists of two parts. The first part collects demographic data while the second part contains four case presentations. These presentations include figures or photographs of several stages of proximal and occlusal lesions of primary molars, and buccal and proximal lesions of primary maxillary incisors. The questionnaire also includes items about restorative treatment criteria, preparation techniques, and the use of restorative materials.

The questionnaire was translated into Hebrew using the "forward–backward translation" method, from English to Hebrew and then into English; followed by a comparison of the English translation to the original version, to locate and bridge translation gaps. After formulating the questionnaire, a pilot study was conducted with five pediatric dentists. They were asked to fill out the questionnaire and answer follow-up questions regarding the clarity of the instructions, any difficulties in completing the items or unclear phrases, and related issues. Based on their feedback, the necessary changes were made to the questionnaire.

### Ethics

The study was approved by the Institutional Human Subjects Ethics Committee (HMO-0345-22) and conforms to the declaration of Helsinki. The committee approved the waiver of a separate consent form. Participants provided informed consent by completing and submitting the questionnaire. Only questionnaires that were fully completed and submitted were included in the study.

### Statistical analysis

Descriptive statistics were used to present the data numerically. Frequency distributions and percentages were used to describe qualitative variables. Means, standard deviations, and minimum and maximum values were calculated for quantitative variables. Chi-square tests were conducted to examine independence between pairs of categorical variables. When significance was achieved, tables of residuals were used to assess associations between the categories examined. If the residual value was positive, the association between the two categories was deemed positive. The larger the residual value, the more dominant the association. All the statistical tests were analyzed for a significance level of p-value < 0.05. Analyses were performed using R software version 4.1.3.

## Results

Of 150 ISDC members who received the questionnaire, 106 opened the link and 46 completed and submitted the questionnaire. Accordingly, the response rate was 30.6%. For the distribution of demographic characteristics, see Table 1.

**Table 1:** Distribution of sociodemographic variables in the sample (N = 46)

Variable	Values	Incidence	Percentage	Mean	SD	Range
Gender	Male	15	33			
	Female	30	65			
	NA	1	2			
Type of clinic	Private	27	59			
	Public	19	41			
Dentist's status	Specialist	29	63			
	Resident	12	26			
	General practitioner	5	11			
Age (years)				54.7	11.2	30–76
Years of experience				18.0	11.9	1–50

SD, standard deviation; NA, not applicable

**Fig. 1:**
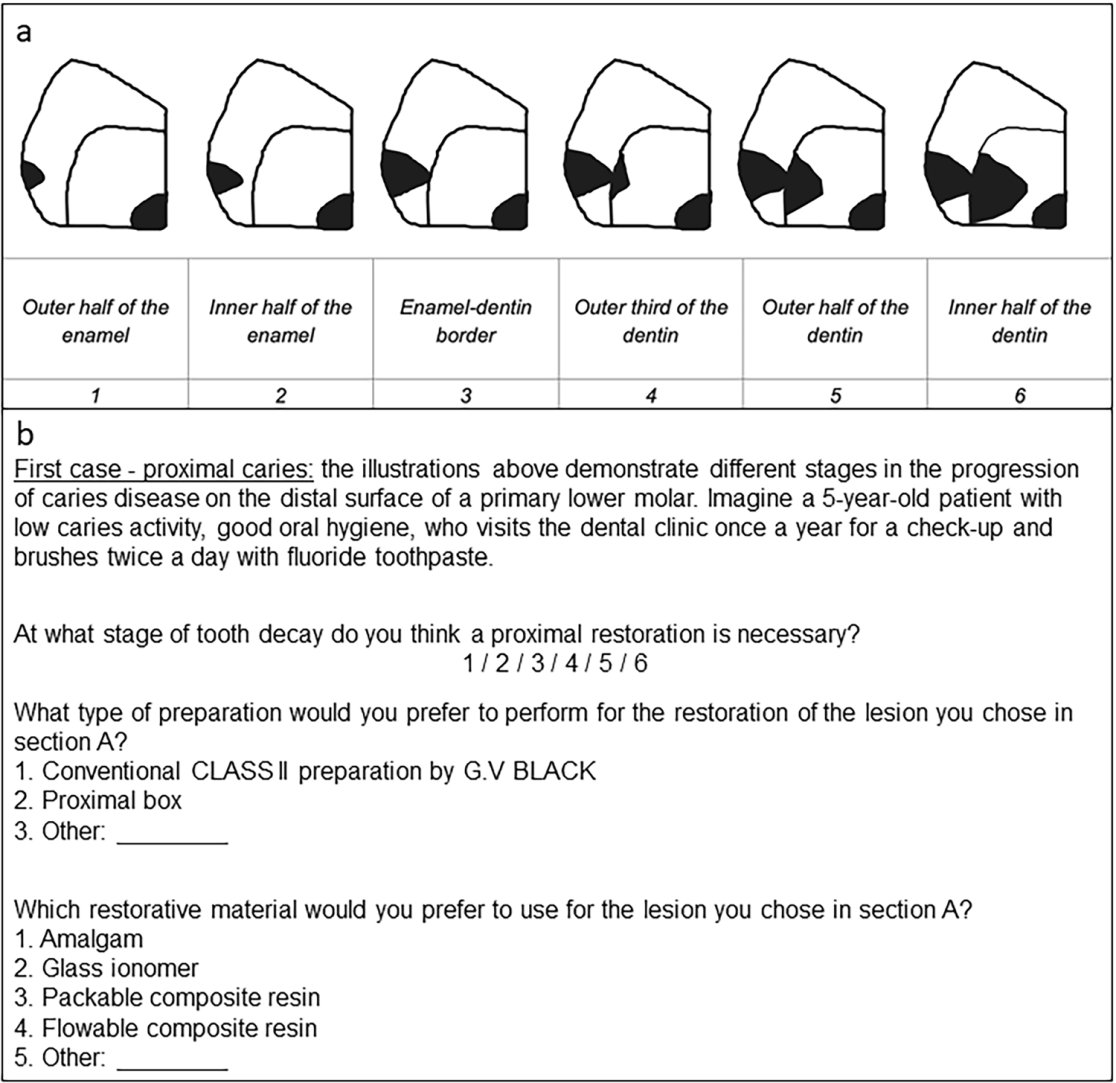
Case presentation of a proximal caries lesion in a primary molar: **a** illustration [from Laske et al 2019] **b** questions

For a proximal caries lesion in the primary molar (Fig. 1), 20 (43.5%) respondents stated they would intervene in stage 3 and 21 (45.5%) stated they would intervene in stage 4 of the lesion. Of these 41 participants, 34 stated that the cavity preparation would be a proximal box (Table 2). A significant association was found between the dentist's status and the type of preparation performed (χ^2^ = 7.3251, df = 2, p-value = 0.025); general practitioners preferring to perform a traditional Class II preparation (Table 3).

**Table 2:** Results of the chi-squared test for independence between the stage of the proximal caries lesion in the primary molar and the study variables

Proximal lesion—primary molar		The stage of caries lesion in which restoration is necessary					Total	Statistical data
		1	2	3	4	5		
Preparation technique	Proximal box	1	1	15	19	2	38	*χ*^*2*^ = 3.8225 *df* = 4 *p-value* = 0.430
	Conventional Class II preparation by G.V Black	0	1	5	2	0	8	
	Total	1	2	20	21	2	46	
The restorative material	Glass ionomer	0	0	1	1	1	3	*χ*^*2*^ = 13.291 *df* = 8 *p-value* = 0.102
	Packable composite resin	0	2	17	16	1	36	
	Flowable composite resin	1	0	2	4	0	7	
	Total	1	2	20	21	2	46

**Table 3:** Table of residuals

Dentist's status	Proximal box	Conventional Class II preparation
Specialist	0.4175016	− 0.90992363
Resident	0.02761841	− 0.06019293
General practitioner	− 1.04826325	2.28463678

Results of the chi-squared test for independence between the type of proximal preparation in the primary molar and the dentist's status

**Fig. 2:**
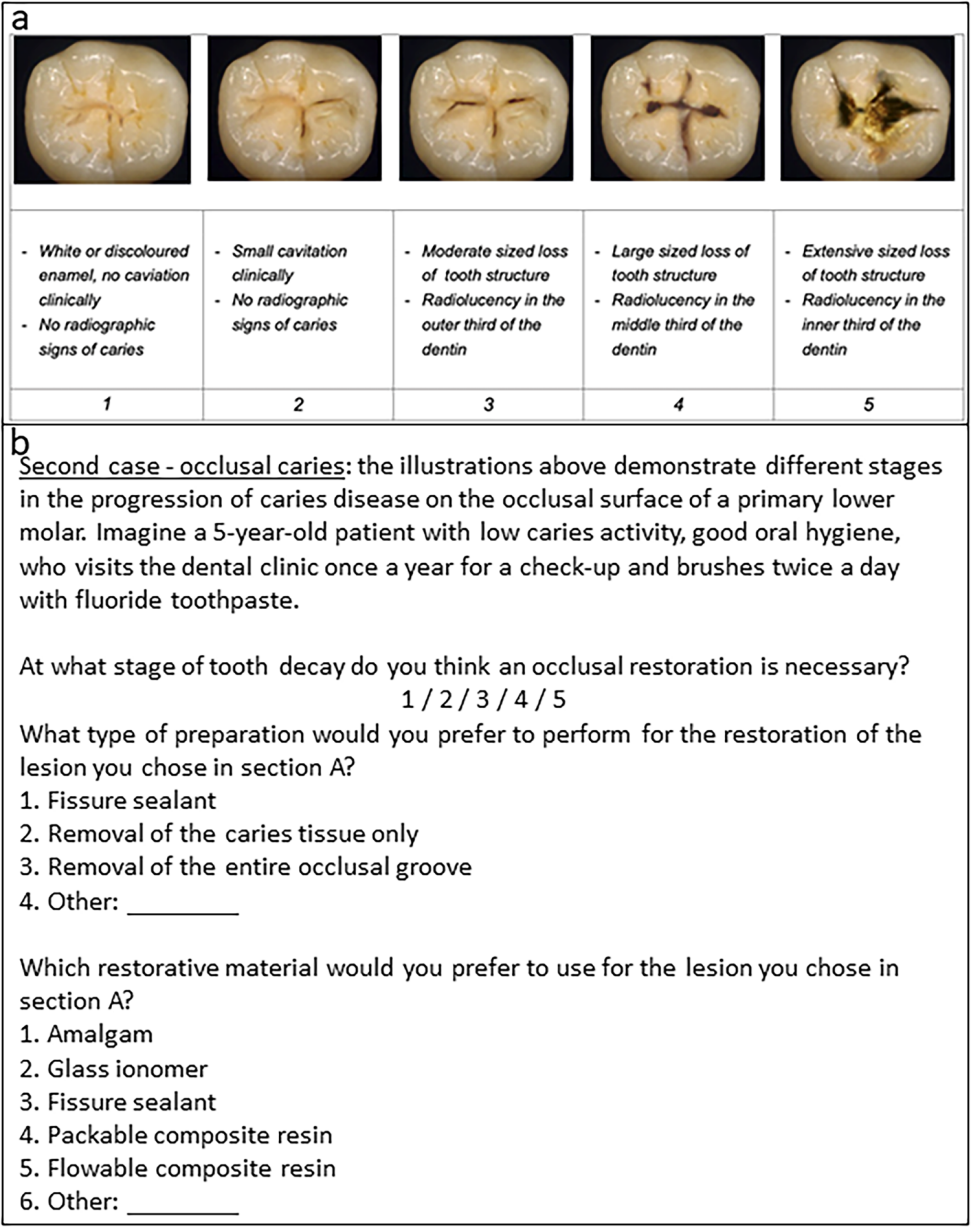
Case presentation of an occlusal caries lesion in a primary molar: **a** illustration [from Laske et al 2019] **b** questions

For an occlusal caries lesion (Fig. 2), 19 (41.3%) respondents stated they would intervene in stage 2 and 18 (39.1%) stated they would intervene in stage 3. Of these 37 participants, 31 would remove only the carious lesion (Table 4). A significant association was found between the stage of caries lesion in which the dentists would intervene and the type of preparation they would perform (*p*-value = 0.004). Accordingly, in an early-stage intervention, preparation for fissure sealant would be performed; while in a later stage of intervention, conventional Class I preparation would be performed (Table 5). A significant association was also found between the stage of caries lesion in which the dentists stated they would intervene and the restorative material they would use (*p*-value = 0.004) (Table 4). At intervention in the early stage, more fissure sealants and flowable composite materials would be used. In the later stages, a packable composite would be applied (Table 6).

**Table 4:** Results of the chi-squared test for independence between the stage of the occlusal caries lesion in the primary molar and the study variables

Occlusal lesion—primary molar		The stage of caries lesion in which restoration is necessary				Total	Statistical data
		1	2	3	4		
Preparation technique	Fissure sealant	1	5	0	0	6	*χ*^*2*^ = 18.653 *df* = 6 *p-value* = 0.004
	Removal of the caries tissue only	0	14	17	6	37	
	Conventional Class I preparation by G.V Black	0	0	1	2	3	
	Total	1	19	18	8	46	
The restorative material	Other material	0	0	0	1	1	*χ*^*2*^ = 28.697 *df* = 12 *p-value* = 0.004
	Fissure sealant	1	3	0	0	4	
	Glass ionomer	0	4	6	1	11	
	Packable composite resin	0	1	8	2	11	
	Flowable composite resin	0	11	4	4	19	
	Total	1	19	18	8	46

**Table 5:** Table of residuals

The stage of caries lesion in which occlusal restoration is necessary	Fissure sealant	Removal of the caries tissue only	Conventional Class I preparation
1	2.407717	− 0.8968544	− 0.255377
2	1.601868	− 0.3280918	− 1.1131624
3	− 1.532262	0.6627381	− 0.1605145
4	− 1.021508	− 0.1713978	2.0465595

Results of the chi-squared test for independence between the stage of occlusal caries lesion in the primary molar and the type of preparation

**Table 6:** Table of residuals

The stage of caries lesion in which occlusal restoration is necessary	Other material	Fissure sealant	Glass ionomer	Packable composite resin	Flowable composite resin
1	− 0.147442	3.0962811	− 0.48901	− 0.48900965	− 0.6426846
2	− 0.6426846	1.0485906	− 0.254969	− 1.66240006	1.1252149
3	− 0.6255432	− 1.2510865	0.817303	1.7813014	− 1.2596942
4	1.9808869	− 0.8340577	− 0.660129	0.06286946	0.3826919

Results of the chi-squared test for independence between the stage of the occlusal caries lesion in the primary molar and restorative material

**Fig. 3:**
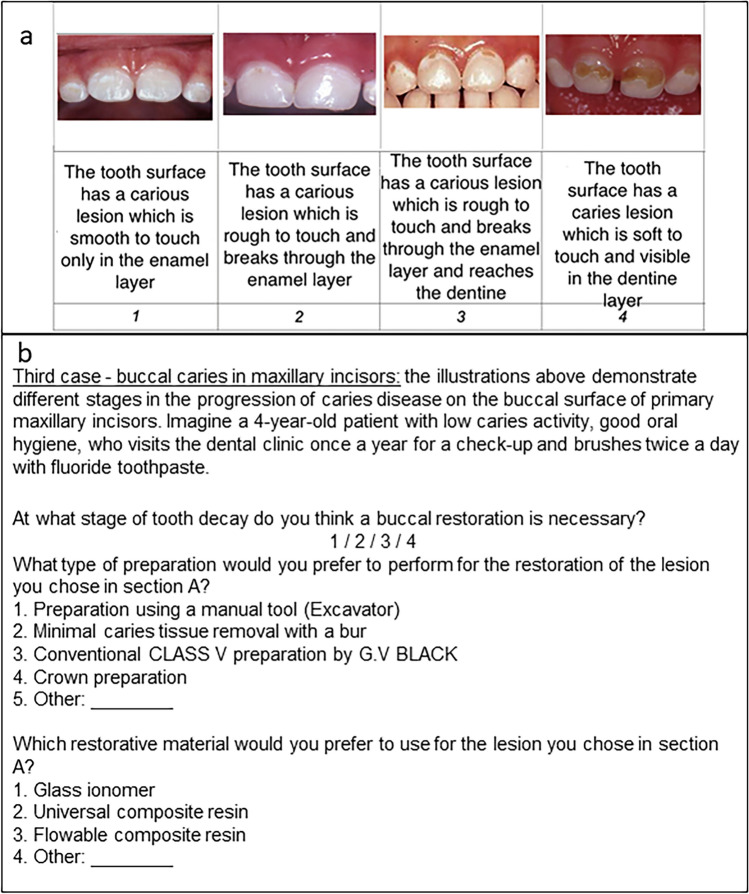
Case presentation of a buccal caries lesion in a primary maxillary incisor: **a** illustration **b** questions

For buccal caries in the primary incisor (Fig. 3), 33 (72%) respondents stated they would intervene at stage 3 and remove minimal caries tissue (Table 7). A clear association was found between the stage of caries lesion in which dentists would intervene and the type of preparation they would choose (*p*-value < 0.001) (Table 7). Intervention at a later stage involves preparation for a crown (Table 8). The results also showed a significant association between the stage of caries lesion at which dentists stated they would intervene and the restorative material they would use (*p*-value < 0.001) (Table 7). Glass ionomer and universal composite resins were preferred for the later stages of the disease (Table 9).

**Table 7:** Results of the chi-squared test for independence between the stage of buccal caries lesion in the primary incisor and study variables

Buccal lesion—primary maxillary incisor		The stage of caries lesion in which restoration is necessary				Total	Statistical data
		1	2	3	4		
Preparation technique	Other preparation	1	0	0	0	1	*χ*^*2*^ = 51.811 *df* = 9 *p-value* < 0.001
	Crown preparation	0	0	4	3	7	
	Conventional Class V preparation by G.V Black	0	0	5	0	5	
	Caries tissue removal with bur	0	4	24	5	33	
	Total	1	4	33	8	46	
The restorative material	Other material	1	0	1	0	2	*χ*^*2*^ = 33.507 *df* = 9 *p-value* < 0.001
	Glass ionomer	0	0	3	3	6	
	Universal composite resin	0	1	12	5	18	
	Flowable composite resin	0	3	17	0	20	
	Total	1	4	33	8	46

**Table 8:** Table of residuals

The stage of caries lesion in which buccal restoration is necessary	Other preparation	Crown preparation	Conventional Class V preparation	Caries tissue removal
1	6.634888	− 0.3900947	− 0.3296902	− 0.84698955
2	− 0.2948839	− 0.7801895	− 0.6593805	0.6673251
3	− 0.8469896	− 0.4559455	0.7460921	0.06701907
4	− 0.4170288	1.6156263	− 0.9325048	− 0.30853045

Results of the chi-square test for independence between the stage of buccal caries lesion in the primary incisor and preparation type

**Table 9:** Table of residuals

The stage of caries lesion in which buccal restoration is necessary	Other material	Glass ionomer	Universal composite resin	Flowable composite resin
1	4.5873171	− 0.3611576	− 0.6255432	− 0.6593805
2	− 0.4170288	− 0.7223151	− 0.4517812	0.9561017
3	− 0.362977	− 0.6286946	− 0.2540839	0.7001788
4	− 0.5897678	1.9153272	1.0566674	− 1.8650096

Results of the chi-square test for independence between the stage of buccal caries lesion in the primary incisor and restorative material

**Fig. 4:**
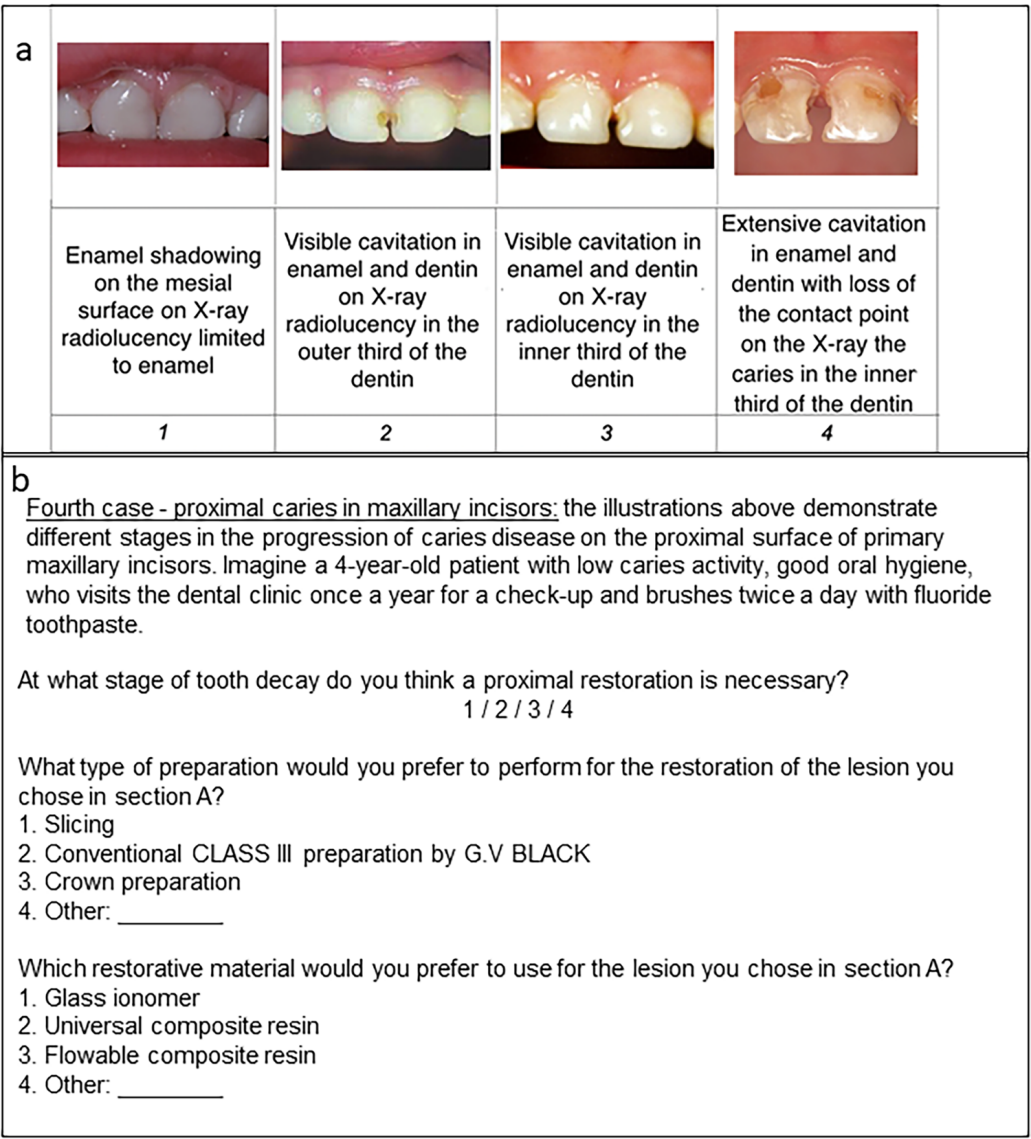
Case presentation of proximal caries in primary maxillary incisors: **a** illustration **b** questions

For a proximal lesion in the primary incisor (Fig. 4), 31 (67%) respondents stated they would intervene in stage 2 of caries. A significant association was found between the stage of caries lesion in which the dentist would intervene and the dentist's status (p-value = 0.026) (Table 10). Accordingly, pediatric dentistry residents would intervene in a later stage (Table 11).

**Table 10:** Results of the chi-squared test for independence between proximal caries lesion stage in the primary incisor and the study variables

Proximal lesion—primary maxillary incisor		The stage of caries lesion in which restoration is necessary				Total	Statistical data
		1	2	3	4		
Preparation technique	Slicing	4	10	3	1	18	*χ*^*2*^ = 10.195 *df* = 6 *p-value* = 0.116
	Conventional Class III preparation by G.V Black	0	7	0	0	7	
	Crown preparation	0	14	5	2	21	
	Total	4	31	8	3	46	
The restorative material	Other: slicing + fluoride	4	8	3	1	16	*χ*^*2*^ = 9.6588 *df* = 9 *p-value* = 0.378
	Glass ionomer	0	1	0	0	1	
	Universal composite resin	0	20	5	2	27	
	Flowable composite resin	0	2	0	0	2	
	Total	4	31	8	3	46	
Expertise status	Specialist	2	24	2	1	29	*χ*^*2*^ = 14.316 *df* = 6 *p-value* = 0.026
	Resident	2	3	5	2	12	
	General practitioner	0	4	1	0	5	
	Total	4	31	8	3	46

**Table 11:** Table of residuals

The stage of caries lesion in which proximal restoration is necessary	Specialist	Resident	General practitioner
1	− 0.3285514	0.9363822	− 0.6593805
2	1.0080802	− 1.7888152	0.3434419
3	− 1.3552054	2.016463	0.1398757
4	− 0.6481049	1.3761249	− 0.5710402

Results of the chi-squared test for independence between the stage of proximal caries lesion in primary incisors and dentists' status

Tables 12 and 13 are macro tables that present the stage of intervention depending on the type of lesion. A significant relation was found between the stage of caries lesion in which the dentist would intervene and the type of lesion (*p*-value < 0.001). For a proximal lesion in the primary molar and a buccal lesion in the primary maxillary incisor, intervention would likely be in the late stage. For an occlusal lesion in a primary molar and a proximal lesion in a primary maxillary incisor, intervention would likely be relatively early.

**Table 12:** Macro table

The stage of caries lesion in which restoration is necessary	Proximal lesion in deciduous molars	Occlusal lesion in deciduous molars	A buccal lesion in deciduous maxillary incisors	Proximal lesion in deciduous maxillary incisors	Statistical data
1	1	1	1	4	*χ*^*2*^ = 83.552 *df* = 12 *p-value* < 0.001
2	2	19	4	31	
3	20	18	33	8	
4	21	8	8	3	
5	2	0	0	0	
Total	46	46	46	46	

Results of the chi-square test for independence between the stage of caries lesion and the type of caries lesion

**Table 13:** Residuals table, macro table

The stage of caries lesion in which restoration is necessary	Proximal lesion in deciduous molars	Occlusal lesion in deciduous molars	Buccal lesion in deciduous maxillary incisors	Proximal lesion in deciduous maxillary incisors
1	− 0.5669467	− 0.5669467	− 0.5669467	1.7008401
2	− 3.2071349	1.3363062	− 2.6726124	4.5434411
3	0.0562544	− 0.3937808	2.9814829	− 2.6439566
4	3.4785054	− 0.6324555	− 0.6324555	− 2.2135944
5	2.1213203	− 0.7071068	− 0.7071068	− 0.7071068

Results of the chi-square test for independence between the stage of caries lesion and the type of caries lesion

## Discussion

Most of the participating dentists stated a preference for a minimal intervention approach, including invasive intervention only at a later stage for patients with a proximal carious lesion in the primary molar. Similarly, Laske et al. (2019) reported that the most common stage for intervention was stage 4. For occlusal lesions in primary molars, the results also collaborate with those of Laske et al.’s (2019), by which stage 3 was the stage most preferred for intervention. However, five (11%) of those who stated they would intervene in stage 2 chose a minimal approach, namely, fissure sealing. This finding illustrates the use of the sealing-in method described by Innes and Manton (2017). We report consensus among our respondents regarding the type of restorative material (flowable composite resin). Flowable composite resin restoration survives very well in shallow Class I lesions (Pinto et al. 2014). It is noteworthy that one participant indicated using a preformed metal crown (Hall Technique) to treat both a stage 4 proximal lesion and a stage 4 occlusal lesion.

Late-invasive intervention was also commonly chosen for buccal lesions in primary incisors, probably after exhausting more conservative and preventive options. The presence of Class V lesions is a main indication for flowable composite resin restorative material. This is mainly due to the mechanical properties of the material and its flow within the cavity, and the excellent level of finish and polish (Baroudi and Rodrigues 2015).

In contrast to the late stage of invasive intervention in the other types of caries lesions, for a proximal carious lesion in primary maxillary incisors, most dentists stated they would intervene invasively relatively early (the second of four stages). Nonetheless, about one-third of the dentists who chose to intervene at this stage chose a slicing type of preparation as a minimal intervention method (Fux-Noy et al. 2023).

The innovation of the current study was the adaptation of the questionnaire for pediatric dentistry. We incorporated specific questions about primary anterior dentition; these lesions are more common in primary than permanent teeth. Early childhood caries refers to caries in the primary teeth of children younger than age 6 years. This caries usually starts in the maxillary primary incisors and is followed sequentially in the first molars, the canines and the second molars, following the eruption pattern of the teeth. Strategies for ECC prevention include reducing *Streptococcus mutans* transmission from caregivers to infants, restricting dietary sugars, tooth brushing, topical fluoride applications and early dental visits (Seow 2018; AAPD 2022). Use of the developed questionnaire can discern attitudes toward more conservative and disease-specific treatment.

The study had several limitations. These include the low number of participants and the low response rate, of about 30%. However, the expected response to an online questionnaire has been described as 25–30% (Fincham 2008). The questionnaire comprised large images, making it difficult to view on mobile devices. Respondents were thus required to use stationary computer screens, and this may have affected the response rate. In addition, the study was conducted on a specific population of dentists, who were all members of ISDC. It therefore does not necessarily represent pediatric dentistry in Israel. Most ISDC members are specialists and residents, and the sample does not represent general practitioners who treat children but are not ISDC members. Finally, there may be a bias in the results due to non-response. Those who chose not to answer the questionnaire may be more likely not to implement a minimal intervention approach. A limitation of the questionnaire developed by Laske et al. (2019) for MID assessment is that it does not address all MID techniques and stages. Specifically, the questionnaire does not include re-orientation and remineralization stages (Innes and Manton 2017), nor repair methods, such as partial caries removal and the Hall technique.

## Conclusions

While the present study has several limitations, it showed that specialists and non-specialists dentists in Israel who treat children tend to use techniques of minimally invasive dentistry. These involve either intervening at a later stage of tooth decay or using conservative methods to remove the caries tissue and restore the tooth. To confirm the results, additional studies on larger samples are needed.

## Data Availability

The data that support the findings of this study are available from the corresponding author upon reasonable request.

## References

[CR1] American Academy of Pediatric Dentistry. Caries-risk assessment and management for infants children and adolescents. In: The reference manual of pediatric dentistry. Chicago: American Academy of Pediatric Dentistry; 2023. p. 301–7.

[CR2] Anil S, Anand PS. Early childhood caries: prevalence, risk factors, and prevention. Front Pediatr. 2017;5:157. 10.3389/fped.2017.00157. 28770188 10.3389/fped.2017.00157 PMC5514393

[CR3] Baroudi K, Rodrigues JC. Flowable resin composites: a systematic review and clinical considerations. J Clin Diagn Res. 2015;9:18–24. 10.7860/JCDR/2015/12294.6129. 10.7860/JCDR/2015/12294.6129 PMC4525629 26266238

[CR4] Berkowitz RJ. Causes, treatment, and prevention of early childhood caries: a microbiologic perspective. J Can Dent Assoc. 2003;69:304–7. 12734024

[CR5] Espelid I, Tveit A, Haugejorden O, Riordan PJ. Variation in radiographic interpretation and restorative treatment decisions on approximal caries among dentists in Norway. Commun Dent Oral Epidemiol. 1985;13:26–9. 10.1111/j.1600-0528.1985.tb00414.x. 10.1111/j.1600-0528.1985.tb00414.x 3855730

[CR6] Espelid I, Tveit AB, Mejare I, Sundberg H, Hallonsten AL. Restorative treatment decisions on occlusal caries in Scandinavia. Acta Odontol Scand. 2001;59:21–7. 10.1080/000163501300035724. 11318041 10.1080/000163501300035724

[CR7] Fincham JE. Response rates and responsiveness for surveys, standards, and the Journal. Am J Pharm Educ. 2008;72:43. 10.5688/aj720243. 18483608 10.5688/aj720243 PMC2384218

[CR8] Fux-Noy A, Goldberg T, Shmueli A, Halperson E, Ram D, Davidovich E, Moskovitz M. Evaluation of proximal slicing in primary maxillary incisors with proximal caries- a retrospective cohort study. BMC Oral Health. 2023;23:904. 10.1186/s12903-023-03648-x. 37990222 10.1186/s12903-023-03648-x PMC10664308

[CR9] Hansen NV, Nyvad B. Non-operative control of cavitated approximal caries lesions in primary molars: a prospective evaluation of cases. J Oral Rehabil. 2017;44:537–44. 10.1111/joor.12508. 28301686 10.1111/joor.12508

[CR10] Hu X, Chen X, Fan M, Mulder J, Frencken JE. What happens to cavitated primary teeth over time? A 3.5-year prospective cohort study in China. Int Dent J. 2013;63:183–8. 10.1111/idj.12028. 23879253 10.1111/idj.12028 PMC9375006

[CR11] Innes NP, Manton DJ. Minimum intervention children’s dentistry - the starting point for a lifetime of oral health. Br Dent J. 2017;223:205–13. 10.1038/sj.bdj.2017.671. 28798464 10.1038/sj.bdj.2017.671

[CR12] Innes NP, Clarkson JE, Speed C, Douglas GV, Maguire A. FiCTION Trial Collaboration. The FiCTION dental trial protocol - filling children’s teeth: Indicated or not? BMC Oral Health. 2013;13:25. 10.1186/1472-6831-13-25. 23725316 10.1186/1472-6831-13-25 PMC3698078

[CR13] Laske M, Opdam N, Bronkhorst EM, Braspenning J, van der Sanden W, Huysmans M, Bruers JJ. Minimally invasive intervention for primary caries lesions: Are dentists implementing this concept? Caries Res. 2019;53:204–16. 10.1159/000490626. 30107377 10.1159/000490626 PMC6425814

[CR14] Pinto GS, Oliveira LJ, Romano AR, Schardosim LR, Bonow ML, Pacce M, Correa MB, Demarco FF, Torriani DD. Longevity of posterior restorations in primary teeth: results from a paediatric dental clinic. J Dent. 2014;42:1248–54. 10.1016/j.jdent.2014.08.005. 25150105 10.1016/j.jdent.2014.08.005

[CR15] Santamaria RM, Innes NP, Machiulskiene V, Evans DJ, Alkilzy M, Splieth CH. Acceptability of different caries management methods for primary molars in a RCT. Int J Paediatr Dent. 2015;25:9–17. 10.1111/ipd.12097. 24602167 10.1111/ipd.12097

[CR16] Seow WK. Early childhood caries. Pediatr Clin North Am. 2018;65:941–54. 10.1016/j.pcl.2018.05.004. 30213355 10.1016/j.pcl.2018.05.004

